# An AluYb8 mobile element characterises the risk haplotype of the TMEM106B locus associated with neurodegeneration

**DOI:** 10.1002/alz.090857

**Published:** 2025-01-03

**Authors:** Alex N Salazar, Niccoló Tesi, Yolande A.L. Pijnenburg, Sven J van der Lee, Lydian Knoop, Sanduni Wijesekera, Jana Krizova, Mikko Hiltunen, Markus Damme, Leonard Petrucelli, Marcel JT Reinders, Marc Hulsman, Henne Holstege

**Affiliations:** ^1^ Amsterdam UMC, Amsterdam Netherlands; ^2^ University of Eastern Finland, Kuopio Finland; ^3^ Christian‐Albrechts‐Universitaet Kiel, Kiel Germany; ^4^ Mayo Clinic, Jacksonville, FL USA; ^5^ Delft University of Technology, Delft Netherlands

## Abstract

**Background:**

The TMEM106B protein is critical for proper functioning of the endolysomal system, which is utilised by all cells to traffic and degrade molecular cargo. Genome‐wide association studies identified a haplotype in the *TMEM106B* gene that is associated with increased risk for Alzheimer’s disease (AD), amyotrophic lateral sclerosis (ALS), and frontotemporal lobar degeneration with TAR DNA binding protein inclusions (FTLD‐TDP). However, the causal variant that drives the association has thus far remained elusive.

**Methods:**

We generated long‐read whole‐genome sequencing data of 256 individuals, primarily from Dutch descent. We characterized SNPs and larger structural variants in the *TMEM106B* locus using *de novo* genome assembly.

**Results:**

We identified an insertion of an AluYb8 retrotransposon in the 3’ UTR of *TMEM106B* gene, that was in complete linkage with the *TMEM106B* risk‐SNP. AluYb8 retrotransposons have the propensity to propagate through our genomes by utilising a ‘copy‐paste’ mechanism, and once integrated can disrupt transcription and translation of nearby genes. However, propagation of retrotransposons can be suppressed by methylation of the insert and its surrounding regions. Indeed, we observed that the risk haplotype with the AluYb8 insertion, but not the protective haplotype, accumulated CpG islands over evolutionary time. Notably, we observed similar retrotransposon insertions in the 3’ UTR of *TMEM106B* orthologs in non‐primate species. This suggests a survival advantage, which may be explained by recent findings that TMEM106B is an entry‐receptor for specific viruses in lung‐tissue, such that SINE‐mediated downregulation of *TMEM106B* may limit viral infection‐load across species.

**Conclusions:**

We speculate that AluYb8‐mediated downregulation of TMEM106B may be protective at younger ages in lung tissues, but that at advanced ages its downregulation in the brain may contribute to increased risk of neurodegenerative diseases. Furthermore, next to the supression of AluYb8 activation by DNA methylation, it may also be suppressed by TDP‐43, in its role in post‐translational RNA‐processing. This leads us to further speculate that age‐related demethylation and age‐related dysregulation of TDP‐43 may result in a negative feedback loop that ultimately reduces the endolysosomal activity in cells. We argue that such a mechanism would explain why increased age is among the strongest risk factors of neurodegenerative diseases.

